# Is it beneficial to use Internet-communication for escaping from boredom? Boredom proneness interacts with cue-induced craving and avoidance expectancies in explaining symptoms of Internet-communication disorder

**DOI:** 10.1371/journal.pone.0195742

**Published:** 2018-04-19

**Authors:** Elisa Wegmann, Sina Ostendorf, Matthias Brand

**Affiliations:** 1 Department of General Psychology, Cognition and Center for Behavioral Addiction Research (CeBAR), University of Duisburg-Essen, Duisburg, Germany; 2 Erwin L. Hahn Institute for Magnetic Resonance Imaging, Essen, Germany; Swansea University, UNITED KINGDOM

## Abstract

The use of online-communication applications including messengers (e.g. WhatsApp) or social networking services (e.g. Facebook) on the smartphone has turned into daily practice for billions of people, for example during waiting times. An increasing number of individuals show diminished control over their usage of these applications despite negative consequences in everyday life. This can be referred to as Internet-communication disorder (ICD). The current study investigated the effect of boredom proneness on symptoms of an ICD. It further examined the mediating role of cognitive and affective mechanisms, namely expectancies to avoid negative feelings online and cue-induced craving. The results of a structural equation model (N = 148) illustrate that boredom proneness is a risk factor for the development and maintenance of an ICD as it had a significant direct effect on ICD symptoms. Furthermore, boredom proneness predicted avoidance expectancies as well as cue-induced craving. Both in turn enhanced the risk of developing ICD tendencies. Moreover, both variables mediated the effect of boredom proneness on ICD and interacted among each other. In summary, the results demonstrate that people who have a higher susceptibility to experience boredom show higher expectancies to avoid negative emotions online, which promotes higher craving reactions when being confronted with specific cues (e.g. an incoming message), and could result in ICD tendencies.

## Introduction

With the launch of the smartphone more than ten years ago, the number of people using it in everyday life is still rising. The number of smartphone users worldwide is forecast to reach 2.32 billion in 2017, and is expected to reach 2.87 billion users in 2020 [1]. Among others, the most popular online applications used on the smartphone are online-communication applications. They allow users to have direct contact with others, to stay connected with distant friends, and to share personal information, pictures, or videos [2, 3]. The term ‘online-communication applications’ includes very popular applications such as the Instant messaging service WhatsApp with more than 1.3 billion active users every month [4] or social networking services such as Facebook with 2 billion monthly active users [5]. Besides many advantages of Internet communication and the smartphone use in general, there is a growing amount of individuals experiencing negative consequences due to an excessive and time-consuming use of these applications [2, 6–8]. Especially the availability of different mobile devices and the easy and permanent access to such applications allow people to interact and communicate with others throughout the day—any time, at any place [9, 10]. This behavior may lead to a pathological and compulsive use, which is comparable to other behavioral addictions or substance-use disorders as suggested by various studies and researchers [7, 8].

### Cognitive and affective correlates of Internet-communication disorder

The increasing use of the Internet all over the world leads research to more and more studies focusing on Internet-use disorder as a specific type of behavioral addiction [2, 7, 11]. Furthermore, some studies suggest a specific type of Internet-use disorder, the Internet-communication disorder (ICD). ICD describes the addictive use of online-communication applications [6–8, 12]. Symptoms of an ICD, which are derived from the characteristics of an Internet-use disorder, are defined as loss of control, relapse, withdrawal symptoms, preoccupation, neglect of interests, tolerance, and negative consequences in social, professional, or personal life [6, 7, 13, 14]. Davis [12] offered the first theoretical model describing the mechanisms of an unspecific pathological use of the Internet as well as of a specific Internet-use disorder. More recently, Brand, Young [7] introduced a new theoretical model, the Interaction of Person-Affect-Cognition-Execution (I-PACE) model, which summarizes potential mechanisms of the development and maintenance of specific Internet-use disorders, such as ICD. The I-PACE model illustrates the interaction of person’s core characteristics as well as affective, cognitive, and executive components. It suggests that person’s core characteristics such as personality, social cognitions, psychopathological symptoms, biopsychological factors, and specific predispositions affect the subjective perception of a situation. This perception is formed by factors such as the confrontation with addiction-related cues, stress, personal conflicts, abnormal mood as well as by individual affective and cognitive responses. The latter include cue-reactivity, craving, attentional bias, or further Internet-related cognitive biases and dysfunctional coping style. These individual affective and cognitive factors are assumed to mediate or moderate the effect of a person’s core characteristics on the development and maintenance of a specific Internet-use disorder. Brand, Young [7] illustrate that the effect of affective and cognitive responses interacts with executive factors, such as inhibitory control. The decision to use a certain application in order to experience gratification or compensation may then lead to an excessive use of that application, thereby reinforcing specific predispositions as well as affective, cognitive, and executive factors similar to a vicious circle (for a more detailed description of the model and a detailed overview of empirical studies, see [7]).

Former studies already showed that the effect of psychopathological symptoms, such as depression and social anxiety, and the effect of personality aspects, such as stress vulnerability, self-esteem, and self-efficacy, on tendencies of an ICD is mediated by specific cognitions, such as a dysfunctional coping style and Internet-use expectancies [8, 15]. Wegmann, Oberst [16] demonstrated that especially avoidance expectancies, including the desire to escape from reality, to distract from real-life problems, or to avoid loneliness, are relevant for explaining ICD symptoms. Brand, Laier [17] as well as Trotzke, Starcke [18] showed that high expectancies towards the usage of specific applications as a possibility to experience pleasure or to distract from problems mediate the relationship between personal aspects and a generalized (unspecific) Internet-use disorder as well as an Internet-shopping disorder, respectively.

In addition to the concept of Internet-use expectancies, Brand, Young [7] further argue that cue-reactivity and craving seem to be important constructs within the development and maintenance of a pathological use of specific applications. This assumption is based on former research about substance-use disorders (see for example results in [19] as well as other behavioral addictions [20], which show that addicts are vulnerable to addiction-related stimuli that trigger reward-processing areas in the brain [21–25]. Craving describes the desire or urge to take drugs or to show an addictive behavior repeatedly [26, 27]. The concept of cue-reactivity and craving has been transferred to the study of behavioral addictions. Behavioral correlates of cue-reactivity and craving have already been observed in Internet-shopping disorder [18], Internet-pornography-viewing disorder [28, 29], Internet-gaming disorder [30, 31], Internet-gambling disorder [32, 33], and ICD [34].

Although studies emphasize the important role of these affective (cue-reactivity and craving) and cognitive (Internet-related expectancies) components in the development and maintenance of a specific Internet-use disorder, the interaction of these factors, which is postulated in the I-PACE model, remains unclear. The current study is based on some main assumptions of the I-PACE model, especially the mediation effects of affective and cognitive mechanisms on the relationship between person’s core characteristics and symptoms of an ICD. The aim of this study is to investigate the effect of person’s core characteristics on ICD mediated by both Internet-related cognitive biases (e.g. Internet-use expectancies) and affective biases (e.g. cue-induced craving). Based on Wegmann, Oberst [16], we assume that the effect of expectancy to avoid negative emotions by using online-communication applications is mediated by cue-induced craving, as described in the model of Brand, Young [7]. As a second aim of the study, we focus on the investigation of the role of the susceptibility to boredom in ICD. Thus, we would like better understanding the relationship between person’s core characteristics and symptoms of a specific Internet-use disorder, which has not yet been investigated in the context of ICD.

### Boredom proneness as a predictor of an ICD

The conceptualization of boredom is determined by different situational and individual factors [35]. Boredom itself could be described as a negative state of mind or inner conflict between an expected and a perceived experience [36, 37]. Brissett and Snow [38] defined boredom as a state of “under-stimulation, under-arousal, and lack of psychological involvement associated with dissatisfaction, and individuals try to cope with boredom by seeking additional stimulation” [39]. This state is also associated with unpleasant feelings, which individuals try to escape from [40, 41]. Merely boredom proneness is defined as trait boredom. The construct of boredom proneness is often “operationalized as an individual’s susceptibility to experience boredom” [35]. Furthermore, boredom proneness includes an individual’s difficulty to draw attention towards a stimulus, to be aware of this attention deficit as well as to try to reduce the experience of boredom as state [35, 42].

Several studies emphasize the clinical relevance of boredom proneness by illustrating that boredom (proneness) is related to alcohol consumption [43], the use of psychoactive substances [44], indexes of depression and anxiety [35], and health problems in general [45]. Zhou and Leung [46] showed that leisure boredom is related to risky behaviors such as delinquency, extreme sensation activity, and drug abuse [36, 46, 47]. As a possible explanation for the relationship between boredom proneness and substance use, (e.g. drinking alcohol), Biolcati, Passini [48] investigated potential mediation effects of expectancies towards the alcohol consumption. The results illustrated that the effect of boredom proneness on binge-drinking behavior is mediated by the expectancies to escape from boredom, to escape from problems, and to cope with negative feelings [48]. Furthermore, empirical research about different behavioral addictions or pathological behaviors explains the relevance of boredom for risky behavior. For example, Blaszczynski, McConaghy [49] showed that individuals with gambling disorder scored higher on boredom measures compared to non-gamblers. Gambling seems to be a possibility for them to avoid or reduce negatives states or moods. This is consistent with the results reported by Fortune and Goodie [50] illustrating that pathological gambling is associated with boredom susceptibility, which is a subscale of the Sensation Seeking Scale Form V by Zuckerman, Eysenck [51].

As described earlier, the usage of smartphones in everyday life results from an easy and permanent access that enables ongoing communication and entertainment [2, 52]. We hypothesize that the possibility to have a lasting stimulation leads to a time-consuming and excessive use of the smartphone and online-communication applications. Likewise, avoiding feelings of boredom seems to be the main motivation to use the Internet [53]. Lin, Lin [37] showed that boredom proneness and a high involvement in the Internet both increase the probability of an Internet-use disorder. The authors emphasize that the Internet seems to be a possibility to seek excitement and pleasure, which raises the level of a pathological use. This is consistent with former research emphasizing the relationship between an Internet-use disorder and higher boredom proneness [54–56]. Zhou and Leung [46] specified this relationship and showed that boredom is a predictor of a pathological use of social networking sites as well as of pathological gaming behavior in social networking services. Elhai, Vasquez [42] illustrated that higher boredom proneness mediates the effect of depression and anxiety on problematic smartphone behavior. Overall, we assume that boredom proneness as trait boredom is a personal risk factor regarding the development of an ICD.

### Summary of the study’s aims

**Fig 1:**
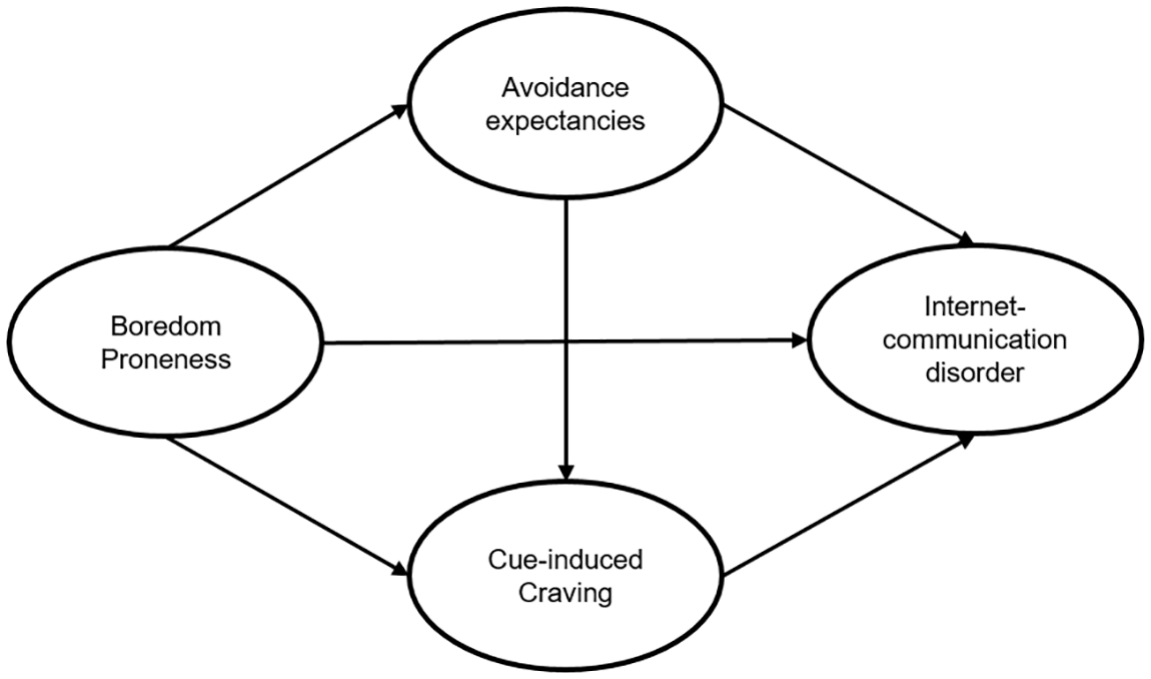
Hypothesized model. The hypothesized model for analyzing the suggested direct and indirect effects including the latent variables of ICD.

The current study aims to contribute to a better understanding of the underlying affective and cognitive mechanisms regarding symptoms of an ICD. Our assumptions are based on previous studies, which reported the effect of boredom proneness on risky behaviors such as substance abuse [57], health risk factors [46], pathological gambling [50], or Internet-use disorder [37, 54]. We assume that individuals who have a higher susceptibility to experience boredom and who repeatedly use the smartphone as a maladaptive coping strategy are more likely to develop a pathological use of online-communication applications. Consistent with the I-PACE model by Brand, Young [7], we hypothesize that the effect of boredom proneness is mediated by specific cognitions. Furthermore and based on the study by Biolcati, Passini [48] we also assume that especially individuals who have a higher boredom proneness as well as expectancies to avoid negative emotions by using online-communication applications experience more negative consequences due to the use of such applications. As a further aim, we investigate the effects of affective and cognitive responses. The I-PACE model suggests that the effect of avoidance expectancies on ICD symptoms is mediated by higher craving experiences. Overall, the mediation effect of cue-induced craving could also be relevant for the mediation effect of avoidance expectancies between boredom proneness and ICD. Fig 1 summarizes the hypotheses in a structural equation model.

## Methods

### Participants and procedure

One hundred forty-eight participants aged between 18 and 60 years (*M* = 25.61, *SD* = 8.94) took part in the current study. Of these, 91 were females and 57 were males. All participants were users of online-communication applications, ranging from two to 19 years of usage (*M* = 8.09, *SD* = 3.09). The online-communication application WhatsApp was the most frequently used application (97.97% of all participants), followed by Facebook (78.38% of all participants), Facebook Messenger (62.84% of all participants), and Instagram (53.38% of all participants). Other online-communication applications such as Twitter, iMessage, Snapchat, or Skype were used by less than 50% of all participants. The participants spend on average 125.41 minutes (*SD* = 156.49) per day using WhatsApp, followed by Instagram (*M* = 57.97, *SD* = 78.76), Snapchat (*M* = 53.71, *SD* = 65.40), and Facebook (*M* = 55.48, *SD* = 84.74). All other applications were used on average less than 30 minutes per day.

We recruited the sample at the University of Duisburg-Essen (Germany) via mailing lists, online social networks, and word-of-mouth recommendations. The study was conducted in a laboratory, individual setting. Firstly, participants were informed in writing about the procedure and gave written consent. We asked them to switch their smartphones to flight mode and to keep it in their pocket during participation. Thereafter, the participants answered online questionnaires and performed a cue-reactivity paradigm as well as further experimental paradigms that are not relevant for the current manuscript. After that, the participants responded to further online questionnaires, such as the Boredom Proneness Scale, the Internet-Use-Expectancies Scale or the short Internet Addiction Test, which will be explained in the following. Overall, the study took about one hour. Students got credit points for their participation. The ethics committee of the University of Duisburg-Essen approved the study.

### Instruments

#### Modified version of the short Internet Addiction Test for Internet-communication disorder (s-IAT-ICD)

Tendencies of an ICD were measured with the short version of the Internet Addiction Test (s-IAT) by Pawlikowski, Altstötter-Gleich [58]. For this study we used the modified version for ICD (s-IAT-ICD) [15]. The scale assesses subjective complaints in everyday life due the use of online-communication applications. At the beginning, a definition of online-communication applications is given. The instructions emphasize that the term online-communication applications includes the active (e.g., writing of new posts) as well as the passive (e.g., browsing and reading new posts) use of social networking sites and blogs such as Facebook, Twitter, and Instagram, as well as Instant messengers such as WhatsApp.

Participants have to rate twelve items on a five-point Likert scale (from 1 = “never” to 5 = “very often”). A sum score was calculated ranging from twelve to 60. Scores > 30 indicate a problematic use of online-communication applications, while scores > 37 indicate a pathological use of online-communication applications. The questionnaire consists of two factors (six items each): loss of control/time management (s-IAT-ICD 1: α = .849) and social problems/craving (s-IAT-ICD 2: α = .708). The overall internal consistency was α = .842. Both factors represent the latent dimension of ICD in the structural equation model.

#### Cue-reactivity and craving

To investigate cue-reactivity and craving, a cue-reactivity paradigm consisting of twelve pictures related to online-communication applications was applied [34, 59]. The visual cues showed different smartphones displaying a conversation through different online-communication applications. The stimuli were pretested and described in a former study by Wegmann, Stodt [34]. In the current study the participants rated each picture regarding arousal, valence, and urge to use the smartphone on a five-point Likert scale (from 1 = “no arousal/valence/urge” to 5 = “high arousal/valence/urge”). Presentation^®^ (Version 16.5, www.neurobs.com) was used for cue presentation and ratings.

Additionally, we used the Desire of Alcohol Questionnaire [60] modified for smartphone-use to assess craving [34]. The questionnaire was presented before and after the cue-reactivity paradigm to measure the baseline craving (DAQ-ICD baseline-craving) as well as potential craving changes after cue exposure (DAQ-ICD post-craving). Therefore, participants had to rate 14 items (e.g., “Using the smartphone would be satisfying right now”) on a seven-point Likert scale (from 0 = “complete disagreement” to 6 = “complete agreement”). After inverting one item, we calculated the mean score [59]. The internal consistencies were α = .851 for DAQ-ICD baseline-craving and α = .919 for DAQ-ICD post-craving. In the following analyses, the DAQ-ICD post-craving and the ratings of the cue-reactivity paradigm were used to represent the latent dimension of the cue-induced craving in the structural equation model.

#### Modified version of the Internet-Use Expectancies Scale for online-communication (IUES)

The Internet-Use Expectancies Scale (IUES) [17] modified for online-communication was used to assess the participants’ expectancies towards the use of online-communication applications [16]. The questionnaire contains two factors (six items each): positive reinforcement (e.g., “I use online-communication applications to experience pleasure”; IUES positive: α = .838) and avoidance expectancies (e.g., “I use online-communication applications to distract myself from problems”; IUES avoidance α = .732). Participants had to rate each item on a six-point Likert scale (from 1 = “completely disagree” to 6 = “totally agree”). Based on former research and theoretical assumptions, only the avoidance expectancies variable was relevant for the following analyses.

#### Short Boredom Proneness Scale (BPS)

The Short Boredom Proneness Scale (BPS) by Struk, Carriere [61] was used to assess trait boredom proneness. The scale consists of eight items (e.g., “It takes more stimulation to get me going than most people”), which had to be rated on a seven-point Likert scale (from 1 = “completely disagree” to 7 = “totally agree”). An overall mean value was calculated. The internal consistency was α = .866.

### Statistical analyses

The statistical analyses were carried out using SPSS 25.0 for Windows (IBM SPSS Statistics, released 2017). We calculated Pearson’s correlations to test bivariate relationships between two variables. The correlations were interpreted in more detail by using effect sizes. Based on Cohen [62], Pearson’s correlation coefficient *r* ≥ .01 indicates a small, *r* ≥ .03 a medium, and *r* ≥ .05 a large effect. The structural equation model (SEM) analyses were computed by using Mplus 6 [63]. To evaluate the model fit of the SEM, we used the standardized root mean square residual (SRMR; values < .08 indicate a good fit with the data), root mean square error of approximation (RMSEA; values < .08 indicate a good and < .10 an acceptable fit with the data), and comparative fit indices (CFI and TLI; values > .90 indicate an acceptable and > .95 indicate a good fit with the data) [64, 65]. We also used the *χ*^*2*^-Test to check if the data derivate from the defined model. As an additional step to reduce measurement errors for the SEM, we used the method of item parceling for variables that are represented as manifest variables. This method allows building the latent dimensions for these variables in the SEM [66, 67]. Therefore, we checked the inter-correlations between the items of each scale and then created two factors for the latent dimensions of the IUES and the BPS.

## Results

### Descriptive values and multivariate statistics

The mean values and standard deviations of all questionnaires as well as the ratings of the cue-reactivity-paradigm can be found in Table 1. The constructed variables of the item parceling are included as additional values. Table 2 shows the bivariate correlations between these variables. Based on the cut-off scores by Pawlikowski, Altstötter-Gleich [58], 23 participants showed a problematic and seven participants showed a pathological use of online-communication applications, which is associated with subjective complaints in everyday life due to the use of these applications and describes symptoms of an ICD.

**Table 1:** Mean values, standard deviations, and range of the scores of the s-IAT-ICD and the applied scales.

Manifest variable	Latent dimension	*M*	*SD*	Range
s-IAT-ICD sum		23.58	8.16	12.00–48.00
s-IAT-ICD 1	ICD	14.09	5.38	6.00–29.00
s-IAT-ICD 2		9.49	3.65	6.00–23.00
Urge to use the smartphone	Cue-induced craving	1.70	0.85	1.00–4.00
DAQ-ICD post-craving		1.14	1.09	0.00–5.50
IUES avoidance expectancies mean		2.68	1.04	1.00–5.50
IUES avoidance expectancies sub 1^+^	Avoidance expectancies	2.64	1.17	1.00–6.00
IUES avoidance expectancies sub 2^+^		2.72	1.17	1.00–6.00
BPS mean score		2.82	1.11	1.00–6.13
BPS sub 1^+^	Boredom proneness	3.02	1.13	1.00–6.25
BPS sub 2^+^		2.62	1.24	1.00–6.00

^+^ new calculated scores of the original factors based on item parceling

**Table 2:** Bivariate correlations between the scores of the s-IAT-ICD and the applied scales.

	2	3	4	5	6	7	8	9	10	11
1. s-IAT-ICD sum	.936**	.855**	.519**	.645**	.540**	.397**	.564**	.626**	.601**	.574**
2. s-IAT-ICD 1		.618**	.442**	.556**	.520**	.384**	.543**	.544**	.528**	.493**
3. s-IAT-ICD 2			.509**	.623**	.440**	.323**	.460**	.597**	.563**	.556**
4. Urge to use smartphone				.706**	.419**	.363**	.383**	.405**	.370**	.389**
5. DAQ-ICD post-craving					.485**	.420**	.444**	.543**	.517**	.502**
6. IUES avoidance expectancies mean						.891**	.891**	.472**	.466**	.421**
7. IUES avoidance expectancies sub 1							.587**	.444**	.414**	.419**
8. IUES avoidance expectancies sub 2								.396**	.415**	.331**
9. BPS mean									.931**	.943**
10. BPS sub 1										.757**
11. BPS sub 2										

* *p* ≤ .050

** *p* ≤ .010

### The structural equation model

**Fig 2:**
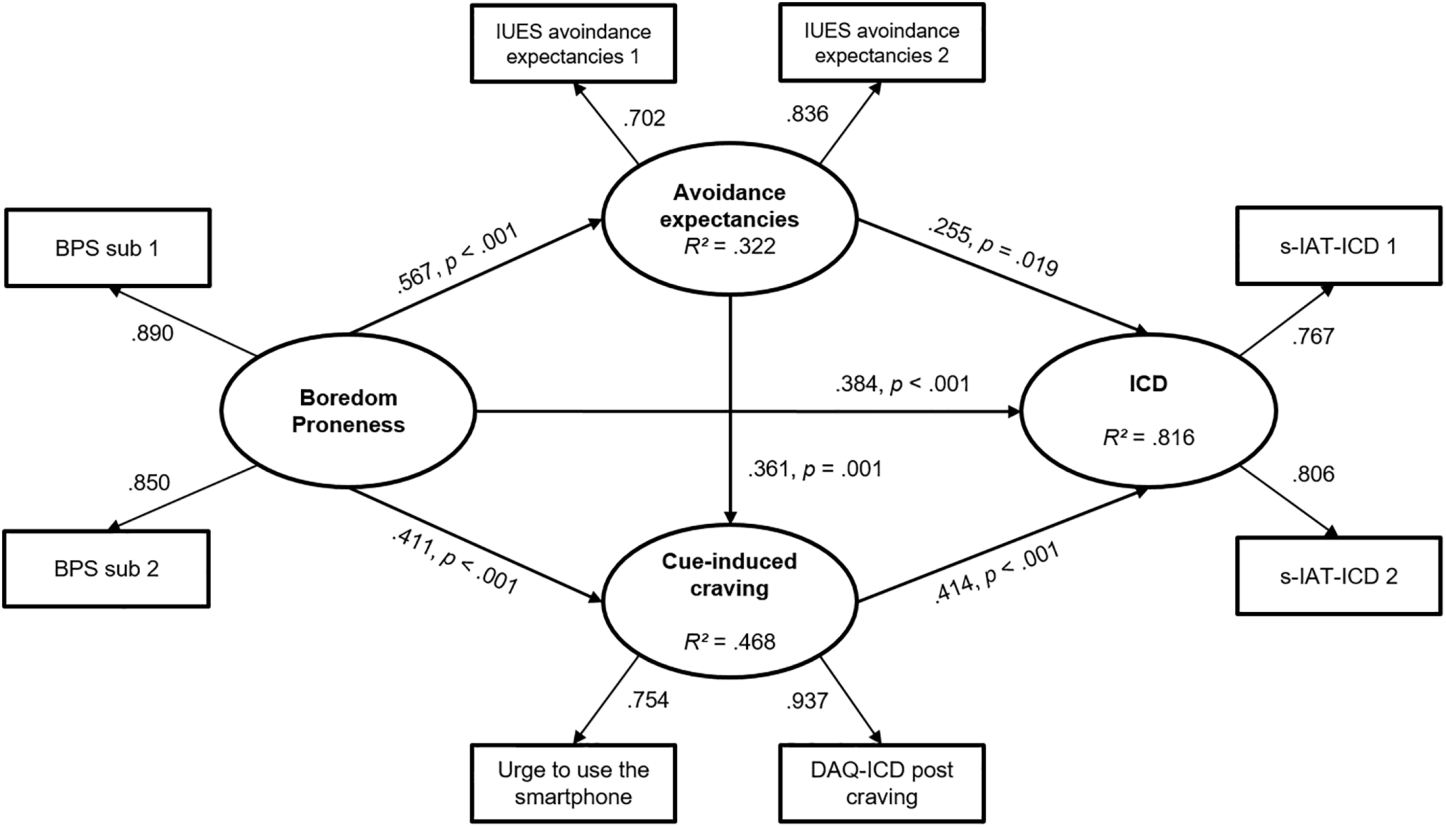
Results of the structural equation model. Results of the structural equation model with ICD as dependent variable including factor loadings on the described latent variables and the accompanying β-weights, *p*-values, and residuals.

The hypothesized structural equation model, on a latent level, showed an excellent fit with the data (SRMR = .029, CFI = .986, TLI = .972, RMSEA = .063, *p* = .299, BIC = 3962.65). The *χ*^*2*^-Test also showed a good fit (*χ*^*2*^ = 22.25, *p* = .074, *χ*^*2*^/df = 1.59). All defined latent dimensions were well represented by the manifest variables used. In the first step, the results indicate that boredom proneness (β = .384, *SE* = .096, *p* ≤ .001), cue-induced craving (β = .414, *SE* = .102, *p* ≤ .001), and avoidance expectancies (β = .255, *SE* = .109, *p* = .011) were significant predictors of ICD tendencies. Boredom proneness also had a direct effect on cue-induced craving (β = .411, *SE* = .100, *p* ≤ .001) and avoidance expectancies (β = .567, *SE* = .084, *p* ≤ .001). Additionally, avoidance expectancies was a significant predictor of cue-induced craving (β = .361, *SE* = .107, *p* = .001). The effect of boredom proneness on symptoms of an ICD was mediated by cue-induced craving (β = .170, *SE* = .058, *p* = .003) and by avoidance expectancies (β = .145, *SE* = .063, *p* = .021). The effect of avoidance expectancies on ICD tendencies was also mediated by cue-induced craving (β = .149, *SE* = .059, *p* = .011). Furthermore, the relationship between boredom proneness and symptoms of an ICD was mediated by avoidance expectancies and, in addition, by cue-induced craving (boredom proneness—avoidance expectancies—cue-induced craving—ICD; β = .085, *SE* = .037, *p* = .021); however this mediation was of small effect only. Overall, the analyzed model significantly explained 81.60% of the variance of ICD symptoms. Fig 2 shows the model with the factor loadings, β-weights, and coefficients.

### Additional analyses

The model described previously was based on theoretical considerations and further empirical evidence such as the structural equation models by Wegmann, Stodt [15] and Wegmann and Brand [8]. Nevertheless, we wanted subsequently controlling the model for other possible influencing factors in order to better understand the underlying mechanisms of an ICD. The first issue we addressed was the close association of boredom proneness with depression and anxiety [35, 68, 69]. A current study by Elhai, Vasquez [42] illustrates that the relationship between psychopathological symptoms and problematic smartphone use is mediated by higher boredom proneness. We assessed psychopathological symptoms such as depression (*M* = 0.53, *SD* = 0.53), interpersonal sensitivity (*M* = 0.72, *SD* = 0.64), and anxiety (*M* = 0.55, *SD* = 0.49) by using the Brief Symptom Inventory Questionnaire by Derogatis [70]. Since the variables operationalizing psychopathological symptoms significantly correlated with the other variables of the current model (all *r*’s ≤ .448, all *p*’s ≤ .024), we included psychopathological symptoms (namely depression, interpersonal sensitivity, and anxiety) as a further latent dimension in the model. Based on the mediation model by Elhai, Vasquez [42] we checked whether the effect of boredom proneness is based on the construct of psychopathological symptoms or whether boredom proneness describes an own statistical increment as it was emphasized in former studies [35, 42, 68].

**Fig 3:**
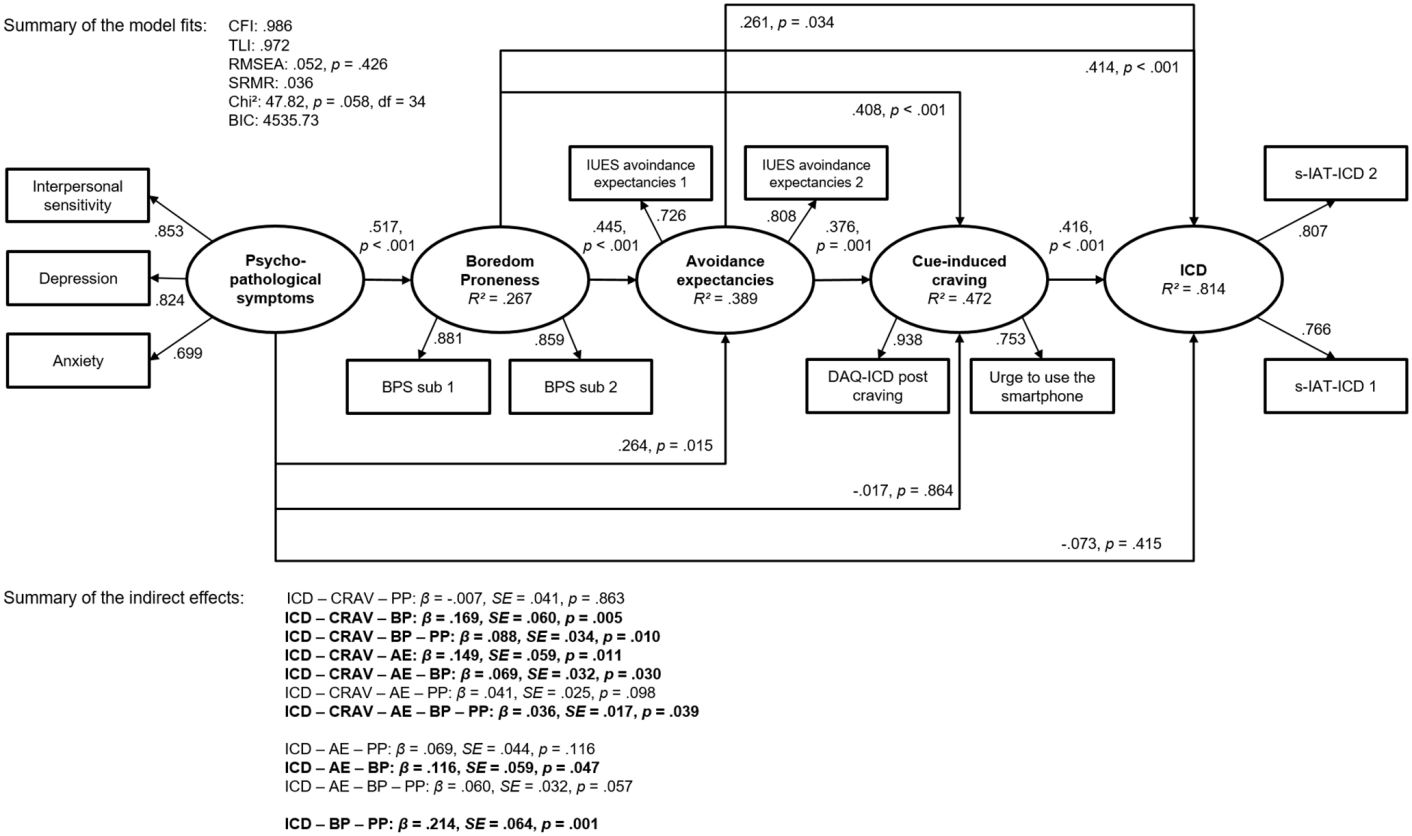
Results of the additional structural equation model. Results of the structural equation model with psychopathological symptoms as further predictor variable including factor loadings on the described latent variables and the accompanying β-weights, *p*-values, and residuals (Abbreviations: PP = psychopathological symptoms, BP = boredom proneness, AE = avoidance expectancies, CRAV = cue-induced craving, ICD = Internet-communication disorder).

As illustrated in Fig 3, the results indicate that psychopathological symptoms play a crucial role in the development and maintenance of an ICD, which is in line with former research [8, 15, 42]. However, the relevance of boredom proneness as an important predictor of symptoms of an ICD is not significantly decreased after including psychopathological symptoms in the structural equation model. This emphasizes that boredom proneness and psychopathological symptoms are related but independent constructs whose effects on tendencies of an ICD are mediated by cognitive and affective components. The results of the additional structural equation model including factor loadings on the described latent variables and the accompanying β-weights, *p*-values, and residuals are summarized in Fig 3.

We also considered age and gender as potential variables which may affect the structure of the current model. Therefore, we first calculated correlations between age and all other variables. The results indicate small correlations (all *r*’s ≤ -.376). These correlations illustrate a familiar pattern that younger participants experience higher subjective complaints in everyday life due to an excessive use of online-communication applications. As a further step, we controlled our data for gender differences by using t-test comparisons for independent samples. The results showed that there was no significant difference between male and female participants (*p* ≥ .319). The structural equation model with additional analysis by gender was calculated using mean structural analysis as a way of proceeding [71]. The fit indices of the structural equation model indicate a good fit with the data (CFI = .975, TLI = .961, SRMR = .060, RMSEA = .075, *p* = .194, BIC = 4050.63). For both male and female participants we found similar result patterns. The female participants showed similar mediation effects as illustrated in the hypothesized structural equation model. For the males, we found no direct effect from avoidance expectancies to tendencies of an ICD (β = .153, *SE* = .133, *p* = .249), no mediation effect of avoidance expectancies on the relationship between boredom proneness and ICD (β = .029, *SE* = .030, *p* = .327), and no mediation effect of craving on the relationship between boredom proneness and symptoms of an ICD (β = .073, *SE* = .065, *p* = .262). Due to the small sample sizes, especially regarding the male sample, the results have to be discussed with caution and should to be controlled in further studies.

## Discussion

In the current study, we tested the validity of a theoretical model assuming interactions between boredom proneness and affective and cognitive components for explaining ICD symptoms. The structural equation model, on latent level, yielded an excellent fit with the data using the method of item parceling to reduce measurement errors. Altogether, boredom proneness and the mediation effects of cognitive and affective components, namely avoidance expectancies and cue-induced craving, explained 81.60% of the variance in ICD symptoms. The results illustrate that boredom proneness has a direct effect on the development and maintenance of an ICD. It was a significant predictor of the expectancies to avoid negative emotions and to escape from reality as well as of cue-induced craving. These affective and cognitive components mediated the effect of boredom proneness on ICD. The results further emphasize the interaction of the mentioned mediators, since the effect of avoidance expectancies on ICD symptoms was partially mediated by cue-induced craving. Furthermore, the mediation of avoidance expectancies on the relationship between boredom proneness and ICD symptoms was mediated by cue-induced craving.

The results support the hypothesis that the relationship between the susceptibility to experience boredom as part of person’s core characteristics, and the experience of negative consequences due to an excessive use of online-communication applications is mediated by affective and cognitive responses to external context-related stimuli, such as visual cues displaying conversations through different online-communication applications. The current results extend the findings of former studies, which already demonstrated that psychopathological symptoms (such as depression or social anxiety) and personality aspects (such as stress vulnerability or self-esteem) have an effect on ICD symptoms, which is mediated by specific cognitions (such as a dysfunctional coping style or Internet-use expectancies) [8, 15]. The results are consistent with the theoretical I-PACE model proposed by Brand, Young [7]. Central to the I-PACE model is the effect of person’s core characteristics in the subjective perception of a situation, e.g. when being confronted with addiction-related stimuli, personal conflicts, or stress. The subjectively colored perception of situational elements leads to individual affective and cognitive responses such as cue-reactivity and craving, which is described as the desire to use a certain application and to reduce negative affective states [20, 24]. The results of the current study support this assumption by showing that participants who have a higher susceptibility to experience boredom (as one of a person’s core characteristics) or to be unable to regulate attention towards stimuli [35], have a higher risk to use online-communication applications excessively. The results are also enhanced by the study by Elhai, Vasquez [42] as well as by our additional analysis, which emphasizes that psychopathological symptoms such as depression, interpersonal sensitivity as well as anxiety could lead to a higher susceptibility of boredom and to a higher risk of a pathological use of online-communication applications. This behavior is reinforced when individuals are confronted with specific (smartphone communication-related) stimuli and experience the desire to use the smartphone or a specific communication application. It seems to be like an automatic habit to use the smartphone after seeing an icon or listening to the sound of an incoming message [34]. Users of online-communication applications might have developed such a habit in order to try to cope with unpleasant feelings like boredom and thus to escape from the experienced under-stimulation [20, 36].

The mediation effect of the avoidance expectancies on the relationship of boredom proneness and ICD symptoms supports this assumption. Similar to cue-induced craving the results demonstrate that the susceptibility to experience boredom leads to expectancies to avoid negative emotions online and to distract from problems by using the smartphone or online-communication applications. This is in line with Biolcati, Passini [48] showing that the relationship between boredom proneness and binge-drinking behavior is mediated by the expectancies to escape from under-stimulation and from reality. The authors assume that especially adolescents, who are more prone to experience boredom in their leisure time, expect to escape from negative emotions by drinking alcohol, which reinforces the risk of binge-drinking behavior [48]. Risky behavior seems to be kind of a maladaptive coping mechanism, where individuals try to find strategies to reduce the propensity of experiencing boredom [35, 39, 40]. The results of Biolcati, Passini [48], Biolcati, Mancini [39], and Harris [40] illustrate main assumptions of the I-PACE model such as the hypothesis that individuals try to escape from negative emotions or to handle abnormal mood especially when being confronted with addiction-related stimuli, which could lead to the decision to use a certain application. Since Zhou and Leung [46] already described the association of boredom proneness with gaming in social networking environments, the current results specify this relation. The experience of gratification or the stimulation in a situation of under-arousal could be described as an important factor that enhances the risk to use certain online applications due to the expectancy to reduce negative affective states in similar situations repeatedly. This is in line with findings of a neuroimaging study by Montag, Markowetz [72] who showed the rewarding aspects of using Facebook via smartphone and higher activation of the ventral striatum when individuals spend time on social networking services.

The second aim of the study was to investigate the interaction of affective and cognitive responses to external stimuli. Former studies already examined the relevance of cue-reactivity and craving [34] as well as Internet-use expectancies [8, 15] and especially avoidance expectancies [16] for the development and maintenance of an ICD. The importance of these two constructs was already shown for specific Internet-use disorders, such as Internet-shopping disorder or pathological buying [18, 59], Internet-pornography-viewing disorder [29], Internet-gaming disorder [30, 73, 74], or generalized (unspecific) Internet-use disorder [17]. To the best of our knowledge, there was no study that investigated the interaction of cue-induced craving and Internet-use expectancies as hypothesized in the I-PACE model [7]. The authors of the I-PACE model assume that Internet-use expectancies predict cue-induced craving, which has an effect on symptoms of a specific Internet-use disorder. Therefore, we hypothesized that cue-induced craving acts as a mediator between Internet-use expectancies (mainly avoidance expectancies) and ICD symptoms. The hypothesis is supported by the current results. The findings indicate that affective and cognitive components interact with each other, which emphasizes key mechanisms of the theoretical model. Individuals with specific Internet-related cognitions (e.g. expectancies to distract from problems, to escape from reality, or to avoid loneliness) seem to be vulnerable to addiction-related cues and seem to experience higher craving-reactions. Regarding the reinforcement mechanisms proposed in the I-PACE model, individuals are assumed to decide to use their “first-choice” applications to distract from this negative state and to experience gratification or compensation. This increases the risk of losing control over the Internet use [7]. The results are a first sign pointing out the interaction between affective and cognitive responses to external and internal stimuli. Since there are further components such as attentional bias and implicit associations as well as the relevance of inhibitory control and executive functions [7], the associations between these factors have to be investigated in further detail. Thereby, future studies should focus on ICD, but also other specific Internet-use disorders.

### Outlook and implications

The usage of smartphones and online-communication applications in every-day life seems to be non-problematic in general. For most individuals it is a common habit to use the smartphone while waiting for another person or for the train for example. Turel and Bechara [75] illustrate the relevance of impulsivity as risk factor of an ICD as well. Overall, online-communication applications seem to be a prime example for the relationship between boredom proneness and a pathological use. It can be assumed that the experience of gratification and compensation by using these applications is a key mechanism regarding the developmental process of an ICD. Although the results are consistent with theoretical assumptions of the I-PACE model by Brand, Young [7], the development of addictive online-communication behavior and ICD symptoms as well as the role of boredom proneness and affective and further cognitive components should be investigated in longitudinal studies. Therefore, more research especially regarding specific reinforcement mechanisms is needed.

Considering this, besides the susceptibility to experience boredom, research should also focus on the subjectively perceived situation. Ben-Yehuda, Greenberg [76] already addressed the relevance of state boredom as a potential risk factor for developing a smartphone addiction, which has to be investigated in further research. This includes the experience of under-stimulation and under-arousal as context-dependent state [38, 57]. It can be assumed that actually perceived boredom is a further relevant explanation why individuals develop the automatic habit to use the smartphone in a situation of under-stimulation. This could be reinforced by the experienced gratification and compensation and therefore increase the probability to use the smartphone in a comparable situation again. So far, further studies should keep in mind that situational factors such as actual mood, personal conflicts, actual experienced boredom, or perceived stress could affect the cognitive and affective components as well as the decision to use a certain application [7, 77].

Given the fact that more and more individuals experience negative consequences in daily life, such as conflicts with family and friends or work-related problems that result from an uncontrolled use of the Internet and its specific applications, there is an increasing need for adequate and guided interventions. In the context of Internet-use disorders and its specific forms, such as ICD, the success of prevention and intervention is assumed to mainly depend on the adequacy of addressing relevant factors. Taking into consideration that personal characteristics may potentially be difficult to modify, interventions should focus on moderating as well as mediating aspects to prevent from an excessive use of certain Internet applications [7]. In this study, expectancies to avoid negative feelings online and cue-induced craving reactions have been emphasized to play a mediating role within the development and maintenance of an ICD. Drawing on specific Internet-use expectancies to change unconducive cognitions could be a first step towards a functional Internet use. People who have trouble to stand boredom or who have a higher susceptibility to experience boredom should be trained to realize that the Internet or the usage of the smartphone is not the only way to cope with daily situations that involve under-stimulation or even unpleasant feelings. This aspect is particularly important because having the expectancy that online-communication applications can foster the escape from real life problems can thereupon promote and intensify craving reactions as shown by the current results, especially when specific stimuli occur. In daily life such stimuli in daily life can be for example seeing other persons using the smartphone or noticing an incoming message. This, in fact, can make it even harder for individuals to resist from the desire to use certain applications. Altogether, individuals can then develop diminished control over their Internet use resulting in negative consequences. Furthermore, approach tendencies towards online-communication applications due to experienced craving should be decreased systematically through training programs that enable individuals to learn how to avoid unregulated reactions to specific stimuli [7]. The effectiveness of common training methods needs further investigation, especially for an ICD.

Finally, we have to mention some limitations. The study was conducted with a convenience sample, which is neither representative for the entire population nor for treatment-seeking patients with an Internet-use disorder. On the basis of the current results, it seems worth investigating the interaction of boredom proneness, craving, and use expectancies in other samples, such as adolescents and treatment-seeking patients. An additional limitation is that we have focused on ICD only. Given that other Internet applications can also be used to escape from boredom or negative feelings, the study should be repeated with samples having other first-choice usages, such as Internet gaming, Internet shopping, or Internet-pornography use.

### Conclusion

The current study aimed to investigate theoretical assumptions regarding the development and maintenance of an ICD. Based on the I-PACE model, the focus was set on mediating effects of cognitive and affective components, namely avoidance expectancies and cue-induced craving, on the relationship between person’s core characteristics and ICD symptoms. This study examined the effect of boredom proneness as a trait variable possibly predicting ICD symptoms. The current results show that boredom proneness could play an important role in ICD. Individuals who have higher susceptibility to experience boredom show higher expectancies to avoid negative feelings by using online-communication applications, which in turn increases negative consequences in daily life. In addition, having avoidance expectancies is associated with a higher experience of craving. This might be due to a potentially higher vulnerability to Internet-communication-related cues, which then makes it even harder not to use online-communication applications. With these results, the underlying mechanisms of an ICD come into shaper relief. Intervention attempts that aim to prevent from an unregulated and excessive use of the Internet and its specific applications can potentially be optimized by considering the concept of boredom proneness and its interaction with cue-reactivity, craving, and expectancies.

## Supporting information

S1 File Dataset_PoNE-D-17-41307R2.sav. This file is the dataset of the current study and contains all variables and information for the conducted analyses.
(SAV)
